# Saliva-Derived Commensal and Pathogenic Biofilms in a Human Gingiva Model

**DOI:** 10.1177/0022034517729998

**Published:** 2017-09-11

**Authors:** J.K. Buskermolen, M.M. Janus, S. Roffel, B.P. Krom, S. Gibbs

**Affiliations:** 1 Department of Oral Cell Biology, Academic Centre for Dentistry Amsterdam (ACTA), University of Amsterdam and Vrije Universiteit Amsterdam, Amsterdam, The Netherlands; 2 Department of Preventive Dentistry, Academic Centre for Dentistry Amsterdam (ACTA), University of Amsterdam and Vrije Universiteit Amsterdam, Amsterdam, The Netherlands; 3 Department of Dermatology, VU University Medical Centre, Amsterdam, The Netherlands

**Keywords:** host-pathogen interactions, oral mucosa, immune evasion, gingivitis, bacteria, keratinocytes

## Abstract

In vitro models that closely mimic human host-microbiome interactions can be a powerful screening tool for antimicrobials and will hold great potential for drug validation and discovery. The aim of this study was to develop an organotypic oral mucosa model that could be exposed to in vitro cultured commensal and pathogenic biofilms in a standardized and scalable manner. The oral mucosa model consisted of a tissue-engineered human gingiva equivalent containing a multilayered differentiated gingiva epithelium (keratinocytes) grown on a collagen hydrogel, containing gingiva fibroblasts, which represented the lamina propria. Keratinocyte and fibroblast telomerase reverse transcriptase–immortalized cell lines were used to overcome the limitations of isolating cells from small biopsies when scalable culture experiments were required. The oral biofilms were grown under defined conditions from human saliva to represent 3 distinct phenotypes: commensal, gingivitis, and cariogenic. The in vitro grown biofilms contained physiologic numbers of bacterial species, averaging >70 operational taxonomic units, including 20 differentiating operational taxonomic units. When the biofilms were applied topically to the gingiva equivalents for 24 h, the gingiva epithelium increased its expression of elafin, a protease inhibitor and antimicrobial protein. This increased elafin expression was observed as a response to all 3 biofilm types, commensal as well as pathogenic (gingivitis and cariogenic). Biofilm exposure also increased secretion of the antimicrobial cytokine CCL20 and inflammatory cytokines IL-6, CXCL8, and CCL2 from gingiva equivalents. This inflammatory response was far greater after commensal biofilm exposure than after pathogenic biofilm exposure. These results show that pathogenic oral biofilms have early immune evasion properties as compared with commensal oral biofilms. The novel host-microbiome model provides an ideal tool for future investigations of gingiva responses to commensal and pathogenic biofilms and for testing novel therapeutics.

## Introduction

The oral microbiome differs in composition among individuals and can contain hundreds of bacterial species (Zaura et al. 2009). In most cases, the oral microbiome does not cause clinical problems and can therefore be considered commensal (Zaura et al. 2009). In contrast, pathogenic oral microbiomes have a detrimental effect on the host tissue, which causes diseases such as gingivitis or caries (Peyyala and Ebersole 2013). To reduce pathogen invasion, host tissue reciprocally interacts with the bacteria by initiating an immune response and secreting antimicrobial peptides (Mans et al. 2009; Ji et al. 2015). To investigate the interactions between the oral microbiome and the host tissue, physiologically relevant models of human oral mucosa are required. Such models can be a powerful screening tool for antimicrobials and hold great potential for drug validation and discovery (Nickerson et al. 2007). In this in vitro study, we investigated the oral host-microbiome interaction using multispecies commensal and pathogenic oral biofilms with an organotypic gingiva model consisting of reconstructed human gingiva epithelium on a fibroblast populated lamina propria (collagen hydrogel).

The complexity of the oral microbiome has been found to have clear effects on the host cell response (Mans et al. 2009; Peyyala and Ebersole 2013). To accurately represent the in vivo situation, the host-microbiome interaction should therefore be investigated with multispecies microbiomes (Klug et al. 2016). This has also been argued by others and is reflected in multiple publications that investigate the host-microbiome interaction with biofilms composed of up to 11 species (Guggenheim et al. 2009; Peyyala et al. 2011; Belibasakis et al. 2013; Bao et al. 2015; Bostanci et al. 2015). Previously, we described the in vitro culture of physiologically relevant complex biofilms derived from the whole salivary microbiome (Janus et al. 2015). These biofilms were cultured from human saliva to phenotypically mimic commensal, gingivitis, or cariogenic biofilms (Janus et al. 2015). Commensal biofilms showed no pathology-related phenotype. In contrast, gingivitis biofilms had increased proteolytic activity, typical for a gingivitis biofilm that is capable of invading the oral mucosa (Bao et al. 2015). Cariogenic biofilms showed increased potential to produce lactate, which lowers the pH and could therefore be capable of dissolving tooth enamel (Marsh 1994).

In addition to physiologically relevant microbiomes, physiologically relevant oral mucosa models are required for the study of the host-microbiome interaction because the complexity of the host tissue also influences the reciprocal host-microbiome relationship (Bao et al. 2015). This is illustrated by the fact that conventional keratinocyte monolayer cultures lack the barrier effect of the multilayered differentiated epithelium, influencing bacterial invasion (Dickinson et al. 2011; Groeger and Meyle 2015). Moreover, crosstalk between cell types (e.g., fibroblasts and keratinocytes) has been reported to synergistically affect inflammatory cytokine secretion in vitro (Spiekstra et al. 2007). To obtain these in vivo–like properties of the oral mucosa in vitro, we developed a 3-dimensional organotypic gingiva model (Buskermolen et al. 2016). The gingiva equivalent consisted of a multilayered differentiating epithelium on a fibroblast-populated collagen hydrogel and closely represented native gingiva.

In the present study. we investigated early host-microbiome interactions after exposing organotypic gingiva equivalents to in vitro grown commensal, gingivitis, and cariogenic oral biofilms for 24 h.

## Materials and Methods

### Commensal, Gingivitis, and Cariogenic Biofilm Culture

Three distinct oral microbiomes (commensal, gingivitis, and cariogenic) were cultured from healthy human saliva as previously described (Janus et al. 2015). The saliva was obtained in accordance with the ethical principles of the 64th World Medical Association Declaration of Helsinki and following procedures approved by the institutional review board of the VU University Medical Centre (Amsterdam, The Netherlands). Briefly, 10 self-reported healthy volunteers donated saliva 24 h after last brushing. The 10 individual saliva samples and a single pooled sample of the 10 donors were diluted 50 times each and used to inoculate 3 types of media to form the commensal, cariogenic, or gingivitis biofilms, as previously described (Janus et al. 2015). To exactly control the number of colony-forming units (CFUs) added on top of the gingiva equivalents and achieve reproducible results, all biofilms were harvested by sonicating (Vibracell VCX130; Sonics & Materials). Thereafter, the number of CFUs per microbiome was determined by serial dilution plating on tryptic soy agar blood plates. The CFUs were counted after 96 h of incubation at 37 °C under anaerobic conditions (Exterkate et al. 2010).

### Microbiome Analysis

To determine the composition of the biofilms cultured from the 10 individual donors and the pooled sample, under commensal, cariogenic, or gingivitis conditions, total DNA isolation, concentration, amplicon sequencing, and data processing and analysis were performed as previously described (Janus et al. 2016). The operational taxonomic units (OTUs) were randomly subsampled at 6,900, and the average abundance of the *duplo* biofilms was calculated for each condition and donor. Sequencing data were used to calculate the Shannon diversity indices. The OTU table was log_2_ transformed, and the data were ordinated by principal component analysis into 2 dimensions via PAST 3.01 software (Hammer et al. 2001).

### Culture and Exposure of Gingiva Equivalents to Oral Microbiomes

Telomerase reverse transcriptase–immortalized human gingiva keratinocyte and fibroblast cell lines were cultured and used for the construction of human gingiva equivalents exactly as previously described, except that no antibiotics were used in the culture media (Buskermolen et al. 2016). The commensal, gingivitis, and cariogenic microbiomes, grown as described from a pool of 10 saliva donors, were diluted in Hank’s Balanced Salt Solution (HBSS) with calcium and magnesium (Gibco) to 10^7^, 10^8^, and 10^9^ CFUs/mL. Each concentration (10 µL) was dripped onto the surface of the gingiva equivalents, for a final exposure of 10^5^, 10^6^, or 10^7^ CFUs/equivalent. Controls were exposed to 10 µL of HBSS. Exposed gingiva equivalents were cultured by air exposure for 24 h at 37 °C, 7.5% CO_2_, and 95% humidity on 1.5 mL of DMEM/Ham’s F12 (3/1; Gibco), supplemented with 1% Fetal Clone III (GE), 0.1μM insulin (Sigma-Aldrich), 1μM isoproterenol (Sigma-Aldrich), 10μM carnitine (Sigma-Aldrich), and 10mM L-serine (Sigma-Aldrich). Each experiment was performed with an intraexperiment duplicate. Three experiments were performed, each with a different batch of gingiva equivalents, which were exposed to different batches of the cultured biofilms, grown independently from the same pool of 10 saliva donors, as described earlier.

### Histology and Fluorescence In Situ Hybridization

Tissue sections (5 µm) were stained with hematoxylin and eosin for histologic examination or processed for immunohistochemistry or fluorescence in situ hybridization (FISH). Immunohistochemistry was performed as previously described (Buskermolen et al. 2016) but with the primary antibody against elafin/SKALP (TRAB2O; Hycult Biotech). To visualize bacteria, the FISH probe EUB338 (5′-GCTGCCTCC CGTAGGAGT-3′) was used according to the manufacturer’s protocol (10-ME-H000; BioVisible). The sections were mounted with a mounting medium containing DAPI (Fluoroshield; Abcam). Histologic evaluation of hematoxylin and eosin, elafin, and FISH was performed by 2 independent scientists on all of the experimental conditions, including the duplicate conditions, of the 3 individual experiments. The microscopic slides were visualized with a fluorescence microscope (Nikon Eclipse 80i microscope with Nikon Plan Fluor 20×/0.50 and 40×/0.75 objectives), followed by contrast enhancement with NIS-Elements software (Nikon Instruments Europe B.V.).

### Protease Activity

To quantify the protease activity in the culture supernatant, fluorescence resonance energy transfer was used as previously described (Kaman et al. 2011; Janus et al. 2015). The culture supernatants of 3 individual experiments, each with an intraexperimental duplicate, of the gingiva equivalents exposed to HBSS (control) and the highest bacterial load (10^7^ CFUs) of each biofilm were measured. Relative fluorescence values were obtained of the gingiva equivalents exposed to the different biofilms against the control gingiva equivalents exposed to HBSS.

### Enzyme-Linked Immunosorbent Assay for Cytokine Production

In accordance with the manufacturer’s specifications, IL-1α, IL-1αRA, IL-4, IL-10, IL-33, IL-6, CCL2, CCL5, CCL20, CXCL8, CXCL12, and thymic stromal lymphopoietin enzyme-linked immunosorbent assays (ELISAs) were performed with the culture supernatants as previously described (Spiekstra et al. 2005). The required antibodies and recombinant proteins were supplied by R&D Systems Inc., except for CXCL8, which was supplied by Sanquin.

### Statistics

The Shannon diversity indices were compared with a 1-way analysis of variance with SPSS. To calculate the significance of the compositional differences among commensal, gingivitis, and cariogenic biofilms and between biofilms from pooled saliva and individual donors per condition, PERMANOVA was performed on the Bray-Curtis similarity index. Groups were considered statistically different if *P* < 0.05. Linear discriminant analysis effect size was used in one-against-all modus (alpha values of 0.05 and LDA threshold of 3.5) to identify the OTUs that differ in relative abundance among the 3 biofilm types (Segata et al. 2011). Cytokine secretions were compared with the Kruskal-Wallis test, followed by Dunn’s multiple-comparison test. Data are represented as mean ± standard error of mean. The number of individual experiments is shown in the figure legends.

## Results

### Differentiating Composition of the Commensal, Gingivitis, and Cariogenic Biofilms

**Figure 1.**
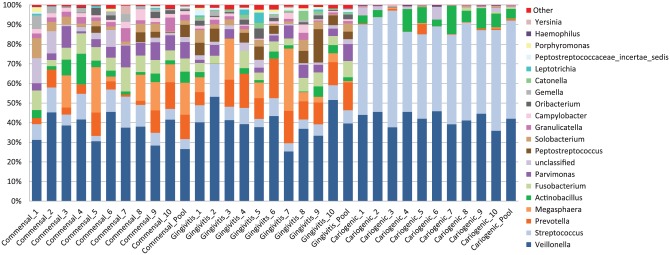
Relative abundance of major bacterial genera. Sequence data for determining the genera are obtained from cultured commensal, cariogenic, and gingivitis biofilms from 10 individual donors and the pooled saliva from the 10 donors. Remaining genera are shown as “other.”

**Figure 2.**
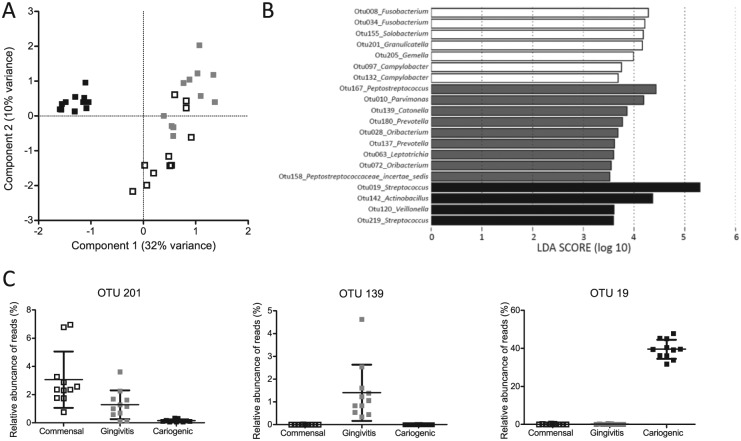
Microbiome analysis of phenotypically different biofilms. (**A**) Principal component analysis plot of commensal (white squares), gingivitis (gray squares), and cariogenic (black squares) biofilms. The data were randomly subsampled and log_2_ transformed. (**B**) Operational taxonomic units (OTUs) that differentiate most among commensal (white bars), gingivitis (gray bars), and cariogenic (black bars) biofilms, ranked by effect size in linear discriminant analysis effect size. (**C**) Box plots of the relative abundance of a typical biomarker detected with linear discriminant analysis effect size for each condition: OTU201 for commensal biofilms, OTU139 for gingivitis biofilms, and OTU19 for cariogenic biofilms. Data represent 11 individually grown biofilms in duplicate for each condition.

The microbial compositions of the human saliva biofilms, which were cultured in such a way as to have commensal, gingivitis, or cariogenic phenotypes, were analyzed by 16S rDNA sequencing. The Shannon diversity index of the cariogenic biofilms (1.6 ± 0.1) was significantly lower than the commensal (2.5 ± 0.2) and gingivitis (2.6 ± 0.3; *P* < 0.001) biofilms, indicating that the composition of the cariogenic biofilms is less diverse than that of the commensal and gingivitis biofilms. The major genera of each biofilm is shown in Figure 1. Sequencing revealed the presence of 70 ± 11 OTUs for the commensal biofilm, 86 ± 12 OTUs for the gingivitis biofilms, and 62 ± 8 OTUs for the cariogenic biofilms (Appendix Table). Principal component analysis of the OTUs clearly separated biofilms with a cariogenic phenotype from biofilms with commensal and gingivitis phenotypes along the first component (Fig. 2A). The commensal and gingivitis biofilm clusters were significantly different (*P* < 0.001, *F* = 3.1) along the second component. Twenty OTUs were found that significantly differentiated the biofilms (Fig. 2B; Appendix Fig.). Typical biomarkers of commensal (*Granulicatella)*, gingivitis (*Catonella* and *Prevotella*), and cariogenic biofilms (*Streptococcus*) were present and corresponded to their phenotype (Fig. 2C; de Soet et al. 1989; Kumar et al. 2005; Sanz et al. 2017). For all conditions, pooled biofilms did not differ in composition or phenotype from biofilms grown from saliva of each donor (*P* = 0.90 for commensal, *P* = 0.81 for gingivitis, *P* = 0.46 for cariogenic). Therefore, the biofilms grown from the pooled saliva were used for the biofilm exposure of the gingiva.

### Gingiva Epithelial Elafin Expression Is Increased during Oral Biofilm Exposure

**Figure 3.**
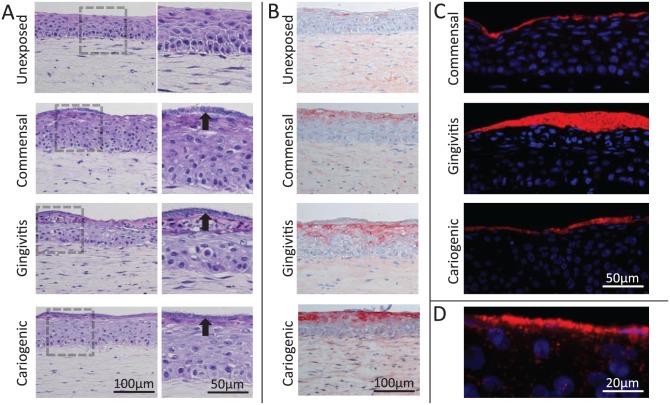
Histology of gingiva equivalents exposed to commensal, gingivitis, and cariogenic biofilms. (**A**) Hematoxylin and eosin staining of unexposed gingiva equivalents and gingiva equivalents exposed to the different biofilms. A biofilm can be seen on top of the epithelium (black arrows). The keratinocytes are enlarged and partly lose the layered organization after biofilm exposure. (**B**) Elafin expression is increased in the upper layers of the epithelium after exposure to the different biofilms. (**C**) The fluorescence in situ hybridization staining of the bacteria (red) shows a thick layer of bacteria on top of the gingiva equivalents. Nuclei are stained with DAPI (blue). (**D**) Enlargement of fluorescence in situ hybridization staining shows localized superficial invasion of the epithelium by bacteria.

The gingiva equivalents consisted of a multilayered differentiated epithelium on a fibroblast-populated collagen hydrogel representing the lamina propria. After a topical exposure for 24 h to commensal, gingivitis, or cariogenic microbiome, a biofilm was clearly seen on top of the gingiva epithelium (Fig. 3A). The organized layered structure of the gingiva equivalents was disrupted particularly in the upper epithelial layers after exposure to the biofilms. Notably, epithelial elafin expression was increased in gingiva equivalents exposed to all 3 types of biofilm (Fig. 3B). FISH showed that the bacteria were predominantly located in a dense layer on top of the gingiva equivalents (Fig. 3C). Furthermore, for all 3 microbiomes, localized invasion into the deeper epithelial layers could be observed (Fig. 3D). There were no clear histologic differences among the gingiva equivalents exposed to the various biofilms based on 3 individual experiments each with an intraexperimental duplicate.

### Protease Activity Is Increased in Gingiva Equivalents Exposed to Biofilms

**Figure 4.**
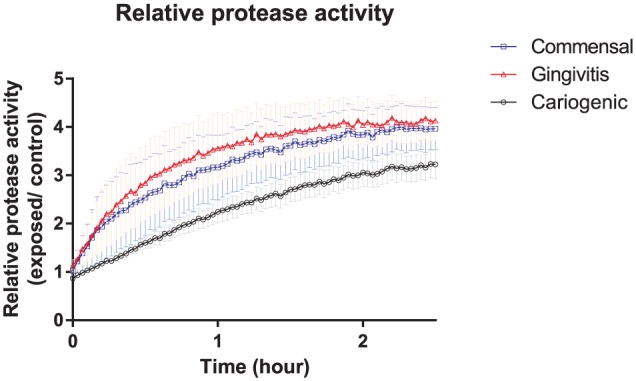
The protease activity of the culture supernatant of gingiva equivalents exposed to 10^7^ colony-forming units of commensal (blue squares), gingivitis (red triangles), or cariogenic (black circles) biofilms was measured by fluorescence resonance energy transfer over 2.5 h and represented relative to unexposed controls. The gingivitis biofilms caused the highest protease activity, followed by the commensal biofilms, and the cariogenic biofilms caused the least protease activity. Data represent the mean ± SEM of 3 individual experiments in duplicate.

The exposure of the gingiva equivalents to the varied biofilms resulted in an increase in protease activity in the supernatant as compared with that of the control gingiva equivalents (Fig. 4). The protease activity in the supernatant was highest for the gingiva equivalents exposed to gingivitis biofilms, followed by commensal biofilms, and was lowest for cariogenic biofilms.

### Commensal Biofilms Trigger a Higher Cytokine Secretion from Gingiva Equivalents Than Gingivitis or Cariogenic Biofilms

**Figure 5.**
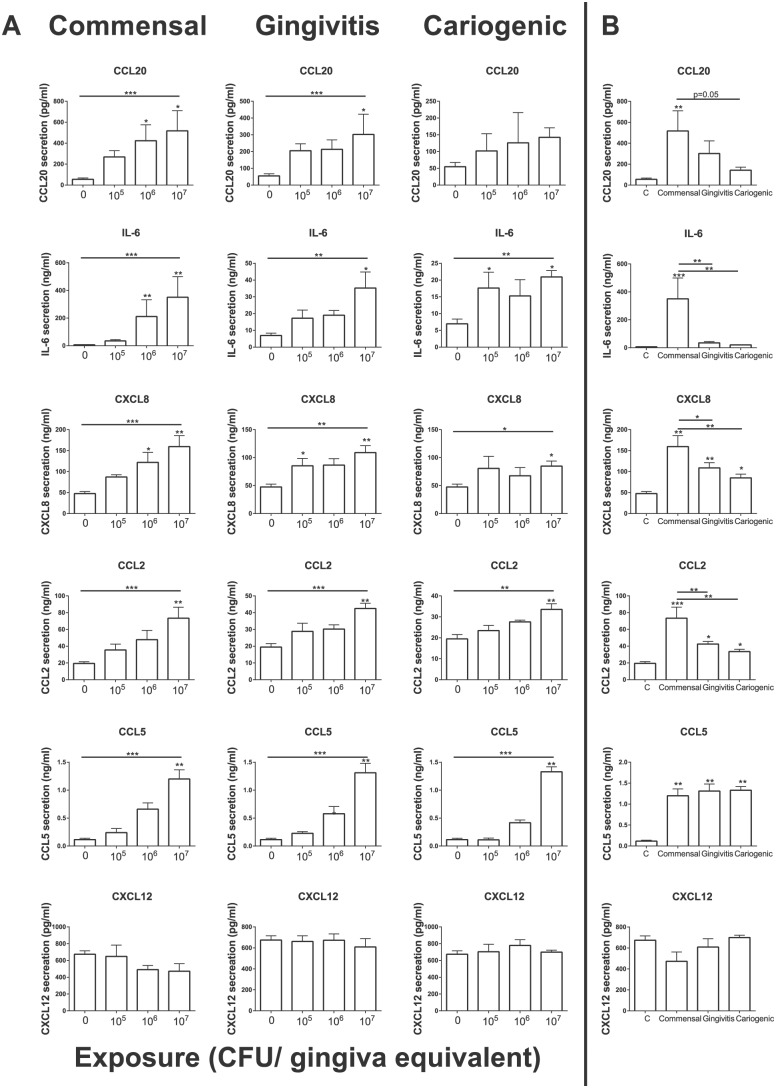
Cytokine secretion by gingiva equivalents after exposure to different biofilms. (**A**) Cytokine secretion (CCL20, IL-6, CXCL8, CCL2, CCL5, CXCL12) of gingiva equivalents after exposure to 0, 10^5^, 10^6^, or 10^7^ colony-forming units (CFU) of the commensal, gingivitis, or cariogenic biofilms. The 3 biofilms show a dose-response for IL-6, CXCL8, CCL2, and CCL5. The secretion of CCL20 was increased after exposure to commensal and gingivitis biofilms but not cariogenic biofilms. CXCL12 secretion was not affected. (**B**) The cytokine secretion by the gingiva equivalents after exposure to 0 (control) or 10^7^ colony-forming units of the different biofilms was compared. CCL20, IL-6, CXCL8, and CCL2 secretion was lower after exposure to the pathogenic biofilms than after exposure to the commensal biofilm. Data represent the mean ± SEM of 3 individual experiments in duplicate as compared by Kruskal-Wallis test (horizontal lines) and Dunn’s multiple-comparison test (stars above columns vs. control). *P < 0.05. **P < 0.01. ***P < 0.001.

To investigate the early innate immune response triggered by the multiple biofilms, cytokines secreted into the gingiva culture supernatant were determined by ELISA (Fig. 5). A significant dose-dependent increase in the secretion of cytokines IL-6, CXCL8, CCL2, and CCL5 was found for gingiva equivalents exposed to commensal, gingivitis, and cariogenic microbiome (Fig. 5A). CCL20 secretion was significantly increased by the cultures exposed to the commensal and gingivitis biofilm but not the cariogenic biofilm. Next, the cytokine secretion by the gingiva equivalents exposed to the highest concentration (10^7^ CFUs) of the varied biofilms was compared (Fig. 5B). Notably, commensal biofilms resulted in the highest increase of CCL20, IL-6, CXCL8, and CCL2 secretion. The secretion of these cytokines was at least 1.5 times higher than those after exposure to the pathogenic gingivitis or cariogenic biofilms. IL-6, CXCL8, and CCL2 secretions were significantly higher after exposure to commensal biofilms than after exposure to gingivitis or cariogenic biofilms. CCL5 was the only cytokine whose secretion was increased by the same amount by all 3 biofilms. CXCL12 and bFGF secretion was not affected by any of the biofilms. IL-1a, IL-1ra, IL-4, IL-10, IL-33, and thymic stromal lymphopoietin secretion was below the detection limit of our ELISAs (data not shown).

## Discussion

Our oral host-microbiome model is the first in vitro model that directly exposes reconstructed human gingiva to physiologically relevant biofilms grown from human saliva. With this model, the early innate inflammatory response to commensal and pathogenic bacteria could be studied in vitro. By using gingiva cell lines, pooling saliva from 10 healthy donors, and growing the biofilms in a standardized way, we have developed a highly reproducible and scalable human test model for investigating host-microbe interactions, which will be of great value for the future study of the oral immune response and disease progression and for testing novel therapeutics aimed at reducing pathogenic biofilm load.

The composition analysis of the phenotypically different biofilms showed that the biofilms contained on average >70 OTUs. This correlates to the in vivo human oral microbiome, which contains between 30 and 300 species of bacteria (Zaura et al. 2009). Therefore, this model is a big improvement on previous multispecies biofilms that contain up to 11 species. Moreover, some species that are known biomarkers for in vivo commensal, gingivitis, or cariogenic biofilms were differentially present in our in vitro models. This correlates to the phenotypical differences observed previously by Janus et al. (2015). The major genera *Veillonella* and *Streptococcus* of the in vitro biofilms are also found in high numbers in vivo (Kumar et al. 2005). Other major in vivo genera, such as *Campylobacter* and *Peptostreptococcus*, were abundantly present in the in vitro grown biofilms. However, a more detailed comparison of the ecology between the in vitro model and the corresponding in vivo niches is not possible per 16S rDNA sequencing. This is due to large species diversity within the in vivo niches.

Analogous with our study, De Ryck et al. (2014) described the use of multispecies biofilms grown from saliva to study the host-microbiome interactions during wound healing. However, in their submerged culture model, there was no direct contact between the biofilm and the host tissue. The direct exposure of the gingiva-equivalent models to the different biofilms resulted in clear histologic changes within the epithelium, which became less organized, particularly in the upper epithelial layers. FISH confirmed the presence of a dense biofilm on top of the gingiva equivalents. To determine the host’s defense against invading bacteria, elafin expression was determined. Elafin is a protease inhibitor that has been reported to have antimicrobial properties both in vitro and in vivo (Williams et al. 2006; Baranger et al. 2008; Verrier et al. 2012). Our finding that elafin expression was increased in the upper epithelial layers suggests that this protein prevents invasion of bacteria into the deeper layers of the epithelium. CCL20 has also been reported to have antimicrobial activity (Yang et al. 2003). Secretion of CCL20 by the gingiva equivalents was increased after the exposure to the commensal and gingivitis biofilms. The increase in elafin and CCL20 indicates a primary host response that combats the bacteria and protects the host tissue integrity.

The activation of the host gingiva tissue was further observed by the increased secretion of proinflammatory cytokines and chemoattractants (IL-6, CXCL8, CCL2, and CCL5). Induction of these cytokines has been associated with periodontal disease in vitro and in vivo (Silva et al. 2007; Peyyala et al. 2012). In our study, the number of CFUs added on top of the gingiva equivalents had a dose-dependent influence on the cytokine secretion. Corresponding to our results, periodontitis severity has been correlated to the cytokine levels in the gingival crevicular fluid (Silva et al. 2007). The correlations between our in vitro model and in vivo data provide evidence of the philological relevance of the model and potential for drug discovery. The chemokines CXCL8, CCL2, and CCL5, which were upregulated in our model, are important in attracting neutrophils, macrophages, and dendritic cells in vivo (Shi and Pamer 2011; Peyyala et al. 2012). Interestingly, the secretion of the cytokines IL-6, CXCL8, and CCL2 by the gingiva equivalents was significantly lower when they were exposed to the pathogenic biofilms than when they were exposed to the commensal biofilms. This could not be explained by the protease activity in the culture supernatants, since the lowest activity was observed for the cariogenic biofilm, which also had the lowest cytokine secretion. Also, the dose-dependent increase of CCL5 secretion was similar after exposure to the different biofilms, making it more likely that specific signals are reduced while others are still released in response to the pathogens. Our results indicate that secretion of specific cytokines was different, depending on whether the biofilm displayed a commensal or pathogenic phenotype. In contrast to these results, inflammatory cytokines in the gingiva crevicular fluid increased during experimental gingivitis in vivo (Scott et al. 2012). These differences may be attributed to the duration of the experiment. Long-term biofilm exposure leads to the eventual destruction of tissue in gingivitis, which increases cytokine release. Our findings represent early innate signals representing the first step in the colonization of healthy tissue by pathogenic bacteria. Our results indicate that pathogenic bacteria reduce inflammatory cytokine levels to allow tissue invasion before the defense mechanisms of the host are activated. Our results are in line with in vitro studies reporting that epithelial cells and neutrophils produce lower levels of inflammatory cytokines in response to periodontal pathogens than to commensal bacteria (Ji et al. 2007; Dickinson et al. 2011; Ji et al. 2015). Although the underlying mechanisms are not fully understood, this may be caused by an early immune evasion mechanism of the pathogenic bacteria (Ji et al. 2007; Dickinson et al. 2011; Bostanci et al. 2015; Ji et al. 2015). Also, it is hypothesized that inflammation induced by commensal bacteria contributes to the control of potential pathogens and thereby maintains gingival health (Dickinson et al. 2011). Our results are in agreement with these ideas and highlight the correlation of our human in vitro model with clinically relevant in vivo data. Therefore, the presented model holds great potential for future research into the interaction between the oral host and microbiome.

## Author Contributions

J.K. Buskermolen, M.M. Janus, contributed to conception, design, data acquisition, analysis, and interpretation, drafted and critically revised the manuscript; S. Roffel, contributed to data acquisition and analysis, drafted the manuscript; B.P. Krom, contributed to conception, design, and data interpretation, critically revised the manuscript; S. Gibbs, contributed to conception, design, and data interpretation, drafted and critically revised the manuscript. All authors gave final approval and agree to be accountable for all aspects of the work.

## Supplementary Material

Supplementary material
