# Supplementary material for: Saliva-Derived Commensal and Pathogenic Biofilms in a Human Gingiva Model
Source: J Dent Res. 2017 Sep 11;97(2):201–8. doi: 10.1177/0022034517729998 (PMC6429568; doi:10.1177/0022034517729998)
Supplement: Supplementary material [file DS_10.1177_0022034517729998.pdf]

Saliva-Derived Commensal and Pathogenic Biofilms in a Human Gingiva Model

J.K. Buskermolen, M.M. Janus, S. Roffel, B.P. Krom, and S. Gibbs

Appendix

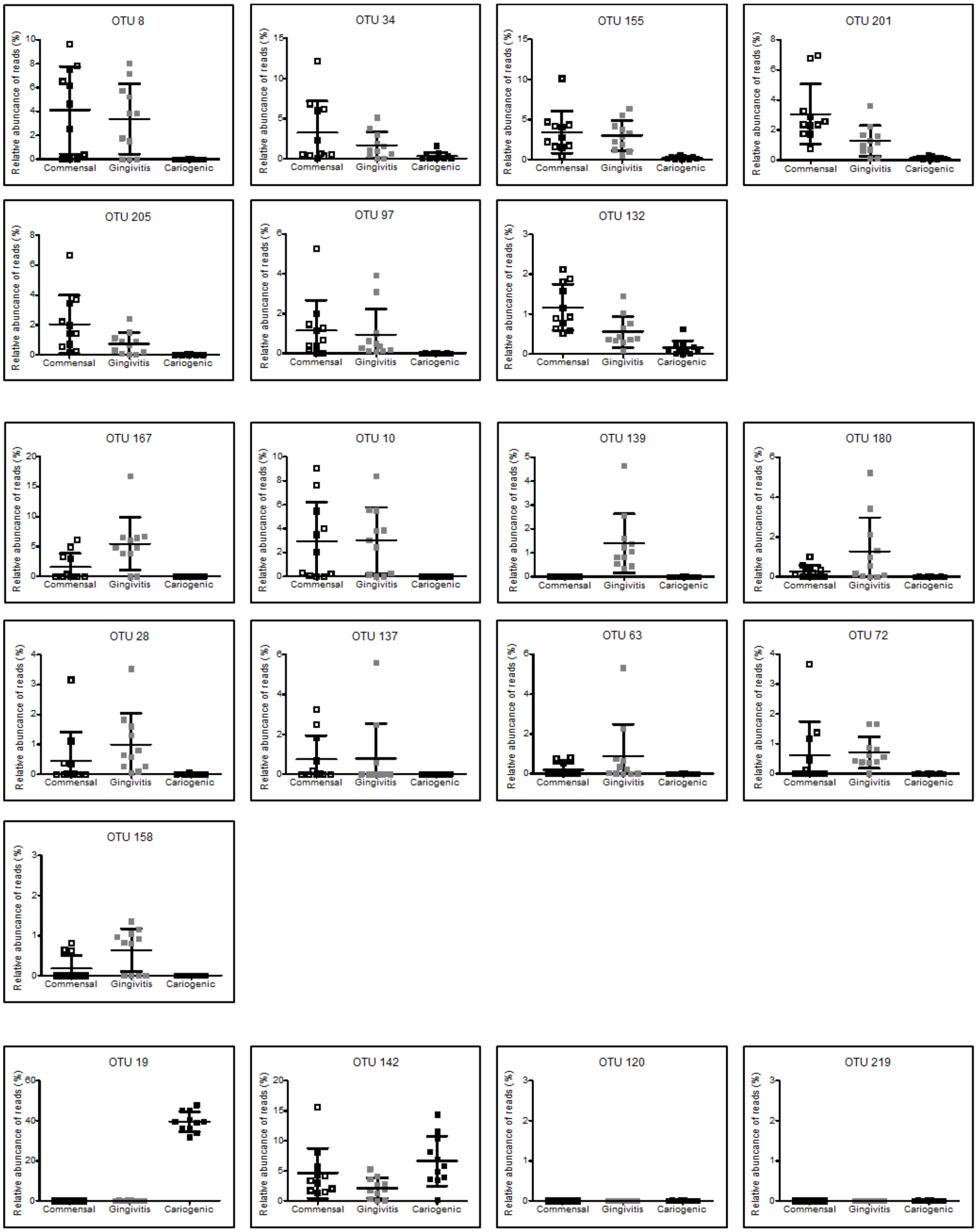

Appendix Figure. Relative abundance of all biomarkers detected with LEfSe at LDA threshold 3.5. A) Biomarkers for commensal biofilms, B) biomarkers for gingivitis biofilms, C) biomarkers for cariogenic biofilms.

Appendix Table

OTU	RDP	Commensal	Gingivitis	Cariogenic
OTU _22	Bacteria; Firmicutes; Negativicutes; Selenomonadales; Veillonellaceae; Veillonella	1092	1937	2323
OTU _19	Bacteria; Firmicutes; Bacilli; Lactobacillales; Streptococcaceae; Streptococcus	2	5	3128
OTU _3	Bacteria; Firmicutes; Negativicutes; Selenomonadales; Veillonellaceae; Veillonella	706	786	558
OTU _142	Bacteria; Proteobacteria; Gammaproteobacteria; Pasteurellales; Pasteurellaceae; Actinobacillus	393	136	340
OTU _54	Bacteria; Firmicutes; Bacilli; Lactobacillales; Streptococcaceae; Streptococcus	93	106	86
OTU _15	Bacteria; Firmicutes; Bacilli; Lactobacillales; Streptococcaceae; Streptococcus	170	50	217
OTU _276	Bacteria; Firmicutes; Negativicutes; Selenomonadales; Veillonellaceae; Megasphaera	1115	48	27
OTU _167	Bacteria; Firmicutes; Clostridia; Clostridiales; Peptostreptococcaceae; Peptostreptococcus	422	264	2
OTU _10	Bacteria; Firmicutes; Clostridia; Clostridiales; Clostridiales_Incertae_Sedis_XI; Parvimonas	20	580	0
OTU _8	Bacteria; Fusobacteria; Fusobacteria; Fusobacteriales; Fusobacteriaceae; Fusobacterium	450	493	0
OTU _17	Bacteria; Firmicutes; Clostridia; Clostridiales; Clostridiales_Incertae_Sedis_XI; Parvimonas	558	11	0
OTU _34	Bacteria; Fusobacteria; Fusobacteria; Fusobacteriales; Fusobacteriaceae; Fusobacterium	7	65	40
OTU _155	Bacteria; Firmicutes; Erysipelotrichia; Erysipelotrichales; Erysipelotrichaceae; Solobacterium	154	289	24
OTU _97	Bacteria; Proteobacteria; Epsilonproteobacteria; Campylobacterales; Campylobacteraceae; Campylobacter	88	44	1
OTU _126	Bacteria; Bacteroidetes; Bacteroidia; Bacteroidales; Prevotellaceae; Prevotella	586	684	23
OTU _90	Bacteria; Proteobacteria; Gammaproteobacteria; Pasteurellales; Pasteurellaceae	152	51	29
OTU _205	Bacteria; Firmicutes; Bacilli; Bacillales; Bacillales_Incertae_Sedis_XI; Gemella	38	2	2
OTU _20	Bacteria; Bacteroidetes; Bacteroidia; Bacteroidales; Prevotellaceae; Prevotella	106	92	21
OTU	Bacteria; Firmicutes; Bacilli; Lactobacillales;	199	81	6

_201	Carnobacteriaceae; Granulicatella			
OTU _132	Bacteria; Proteobacteria; Epsilonproteobacteria; Campylobacterales; Campylobacteraceae; Campylobacter	44	21	15
OTU _137	Bacteria; Bacteroidetes; Bacteroidia; Bacteroidales; Prevotellaceae; Prevotella	48	0	0
OTU _182	Bacteria; Bacteroidetes; Bacteroidia; Bacteroidales; Prevotellaceae; Prevotella	0	0	0
OTU _28	Bacteria; Firmicutes; Clostridia; Clostridiales; Lachnospiraceae; Oribacterium	26	111	0
OTU _139	Bacteria; Firmicutes; Clostridia; Clostridiales; Lachnospiraceae; Catonella	0	58	0
OTU _35	Bacteria; Firmicutes; Bacilli; Lactobacillales; Streptococcaceae; Streptococcus	0	15	0
OTU _70	Bacteria; Bacteroidetes; Bacteroidia; Bacteroidales; Porphyromonadaceae; Porphyromonas	4	6	0
OTU _63	Bacteria; Fusobacteria; Fusobacteria; Fusobacteriales; Leptotrichiaceae; Leptotrichia	0	25	0
OTU _72	Bacteria; Firmicutes; Clostridia; Clostridiales; Lachnospiraceae; Oribacterium	32	55	0
OTU _29	Bacteria; Firmicutes; Bacilli; Lactobacillales; Streptococcaceae; Streptococcus	63	237	1
OTU _128	Bacteria; Bacteroidetes; Bacteroidia; Bacteroidales; Prevotellaceae; Prevotella	0	0	0
OTU _158	Bacteria; Firmicutes; Clostridia; Clostridiales; Peptostreptococcaceae; Peptostreptococcaceae_incertae_sedis	44	58	0
OTU _180	Bacteria; Bacteroidetes; Bacteroidia; Bacteroidales; Prevotellaceae; Prevotella	13	11	2
OTU _14	Bacteria; Actinobacteria; Actinobacteria; Actinomycetales; Actinomycetaceae; Actinomyces	10	7	2
OTU _119	Bacteria; Firmicutes; Clostridia; Clostridiales; Eubacteriaceae; Eubacterium	0	27	0
OTU _53	Bacteria; Proteobacteria; Betaproteobacteria; Neisseriales; Neisseriaceae	1	16	0
OTU _240	Bacteria; Bacteroidetes; Bacteroidia; Bacteroidales; Prevotellaceae; Prevotella	1	10	1
OTU _268	Bacteria; Firmicutes; Negativicutes; Selenomonadales; Veillonellaceae	0	70	0
OTU _133	Bacteria; Proteobacteria; Betaproteobacteria; Neisseriales; Neisseriaceae; Neisseria	0	0	1
OTU _106	Bacteria; Firmicutes; Clostridia; Clostridiales; Lachnospiraceae; Syntrophococcus	0	27	1
OTU _198	Bacteria; Firmicutes; Bacilli; Lactobacillales; Streptococcaceae; Streptococcus	0	0	11

OTU _77	Bacteria; Actinobacteria; Actinobacteria; Coriobacteriales; Coriobacteriaceae; Atopobium	13	6	1
OTU _23	Bacteria; Firmicutes; Negativicutes; Selenomonadales; Veillonellaceae; Veillonella	0	0	13
OTU _185	Bacteria; Firmicutes; Negativicutes; Selenomonadales; Veillonellaceae; Dialister	0	0	0
OTU _190	Bacteria; Bacteroidetes; Bacteroidia; Bacteroidales; Prevotellaceae; Prevotella	0	0	0
OTU _115	Bacteria; Bacteroidetes; Bacteroidia; Bacteroidales; Prevotellaceae	2	2	0
OTU _181	Bacteria; Bacteroidetes; Bacteroidia; Bacteroidales; Prevotellaceae; Prevotella	0	0	0
OTU _43	Bacteria; Bacteroidetes; Bacteroidia; Bacteroidales; Prevotellaceae; Prevotella	7	0	1
OTU _184	Bacteria; Firmicutes; Bacilli; Lactobacillales; Streptococcaceae; Streptococcus	18	47	0
OTU _21	Bacteria; Firmicutes; Bacilli; Lactobacillales; Streptococcaceae; Streptococcus	0	0	1
OTU _263	Bacteria; Firmicutes; Negativicutes; Selenomonadales; Veillonellaceae; Dialister	0	0	0
OTU _84	Bacteria; Proteobacteria; Gammaproteobacteria; Pasteurellales; Pasteurellaceae	9	5	3
OTU _300	Bacteria; Bacteroidetes; Bacteroidia; Bacteroidales; Prevotellaceae; Prevotella	80	90	0
OTU _141	Bacteria; Bacteroidetes; Bacteroidia; Bacteroidales; Prevotellaceae; Prevotella	0	33	0
OTU _47	Bacteria; Bacteroidetes; Bacteroidia; Bacteroidales; Prevotellaceae; Prevotella	0	0	0
OTU _151	Bacteria; Proteobacteria; Gammaproteobacteria; Pasteurellales; Pasteurellaceae; Haemophilus	8	3	1
OTU _202	Bacteria; Bacteroidetes; Bacteroidia; Bacteroidales; Prevotellaceae; Prevotella	0	23	0
OTU _226	Bacteria; Bacteroidetes; Bacteroidia; Bacteroidales	8	6	1
OTU _57	Bacteria; Bacteroidetes; Bacteroidia; Bacteroidales; Prevotellaceae; Prevotella	0	28	0
OTU _307	Bacteria; Proteobacteria; Gammaproteobacteria; Enterobacteriales; Enterobacteriaceae; Yersinia	30	11	0
OTU _280	Bacteria; Firmicutes; Clostridia; Clostridiales; Clostridiales_Incertae_Sedis_XIII; Mogibacterium	0	30	0
OTU _146	Bacteria; Firmicutes; Clostridia; Clostridiales; Lachnospiraceae	0	2	1
OTU	Bacteria; Bacteroidetes; Bacteroidia;	0	6	0

_290	Bacteroidales; Prevotellaceae; Paraprevotella			
OTU _189	Bacteria; Firmicutes; Clostridia; Clostridiales; Lachnospiraceae	0	0	5
OTU _186	Bacteria; Bacteroidetes; Bacteroidia; Bacteroidales; Prevotellaceae; Prevotella	0	2	0
OTU _9	Bacteria; Firmicutes; Bacilli; Lactobacillales; Streptococcaceae; Streptococcus	0	0	0
OTU _73	Bacteria; Firmicutes; Bacilli; Lactobacillales; Streptococcaceae; Streptococcus	0	0	5
OTU _2	Bacteria; Firmicutes; Negativicutes; Selenomonadales; Veillonellaceae; Veillonella	2	4	1
OTU _6	Bacteria; Firmicutes; Negativicutes; Selenomonadales; Veillonellaceae; Veillonella	7	1	0
OTU _86	Bacteria; Firmicutes; Negativicutes; Selenomonadales; Veillonellaceae; Veillonella	14	0	1
OTU _194	Bacteria; Bacteroidetes; Bacteroidia; Bacteroidales; Prevotellaceae; Prevotella	0	0	0
OTU _475	Bacteria; Firmicutes; Clostridia; Clostridiales; Peptostreptococcaceae; Peptostreptococcaceae_incertae_sedis	0	39	0
OTU _56	Bacteria; Firmicutes; Bacilli; Lactobacillales; Streptococcaceae; Streptococcus	4	3	1
OTU _27	Bacteria; Firmicutes; Negativicutes; Selenomonadales; Veillonellaceae; Veillonella	2	0	0
OTU _75	Bacteria; Proteobacteria; Gammaproteobacteria; Pasteurellales; Pasteurellaceae; Haemophilus	0	14	0
OTU _11	Bacteria; Fusobacteria; Fusobacteria; Fusobacteriales; Fusobacteriaceae; Fusobacterium	1	1	0
OTU _266	Bacteria; Firmicutes; Clostridia; Clostridiales; Lachnospiraceae	0	0	3
OTU _162	Bacteria; Firmicutes; Clostridia; Clostridiales; Lachnospiraceae; Oribacterium	0	0	0
OTU _93	Bacteria; Firmicutes; Bacilli; Lactobacillales; Streptococcaceae; Streptococcus	1	0	0
OTU _80	Bacteria; Firmicutes; Clostridia; Clostridiales; Lachnospiraceae; Oribacterium	0	2	0
OTU _341	Bacteria; Firmicutes; Negativicutes; Selenomonadales; Veillonellaceae; Dialister	0	8	0
OTU _74	Bacteria; Firmicutes; Negativicutes; Selenomonadales; Veillonellaceae; Selenomonas	0	0	0
OTU _211	Bacteria; Firmicutes; Clostridia; Clostridiales	2	6	0
OTU _254	Bacteria; Actinobacteria; Actinobacteria; Actinomycetales; Actinomycetaceae;	3	4	0

	Mobiluncus			
OTU _5	Bacteria; Bacteroidetes; Bacteroidia; Bacteroidales; Prevotellaceae; Prevotella	5	5	0
OTU _12	Bacteria; Fusobacteria; Fusobacteria; Fusobacteriales; Fusobacteriaceae; Fusobacterium	4	3	0
OTU _1	Bacteria; Firmicutes; Negativicutes; Selenomonadales; Veillonellaceae; Veillonella	0	0	0
OTU _87	Bacteria; Bacteroidetes; Bacteroidia; Bacteroidales; Prevotellaceae; Prevotella	0	0	0
OTU _58	Bacteria; Firmicutes; Bacilli; Lactobacillales; Streptococcaceae; Streptococcus	0	0	0
OTU _252	Bacteria; Firmicutes; Clostridia; Clostridiales	0	0	0
OTU _223	Bacteria; Actinobacteria; Actinobacteria; Actinomycetales; Actinomycetaceae; Actinomyces	1	0	0
OTU _156	Bacteria; Bacteroidetes; Flavobacteria; Flavobacteriales; Flavobacteriaceae; Capnocytophaga	0	0	0
OTU _188	Bacteria; Firmicutes; Negativicutes; Selenomonadales; Veillonellaceae; Anaeroglobus	0	0	0
OTU _4	Bacteria; Fusobacteria; Fusobacteria; Fusobacteriales; Fusobacteriaceae; Fusobacterium	2	2	0
OTU _32	Bacteria; Firmicutes; Bacilli; Lactobacillales; Streptococcaceae; Streptococcus	0	0	0
OTU _94	Bacteria; Firmicutes; Clostridia; Clostridiales; Clostridiales_Incertae_Sedis_XI; Parvimonas	0	1	0
OTU _131	Bacteria; Bacteroidetes; Bacteroidia; Bacteroidales; Prevotellaceae; Prevotella	6	2	0
OTU _147	Bacteria; Bacteroidetes; Bacteroidia; Bacteroidales; Porphyromonadaceae; Tannerella	0	0	0
OTU _82	Bacteria; Proteobacteria; Gammaproteobacteria; Pasteurellales; Pasteurellaceae; Haemophilus	0	1	2
OTU _24	Bacteria; Firmicutes; Negativicutes; Selenomonadales; Veillonellaceae	3	0	0
OTU _145	Bacteria; Firmicutes; Negativicutes; Selenomonadales; Veillonellaceae; Veillonella	0	0	1
OTU _26	Bacteria; Firmicutes; Negativicutes; Selenomonadales; Veillonellaceae; Veillonella	1	3	0
OTU _352	Bacteria; Firmicutes; Bacilli; Bacillales; Staphylococcaceae; Staphylococcus	0	0	0
OTU	Bacteria; Firmicutes; Bacilli; Lactobacillales;	0	1	0

_18	Streptococcaceae; Streptococcus			
OTU _247	Bacteria; Bacteroidetes; Bacteroidia; Bacteroidales; Prevotellaceae; Prevotella	5	1	0
OTU _164	Bacteria; Actinobacteria; Actinobacteria; Actinomycetales; Actinomycetaceae; Actinomyces	0	3	0
OTU _176	Bacteria; Firmicutes; Clostridia; Clostridiales; Peptostreptococcaceae; Peptostreptococcus	3	1	0
OTU _333	Bacteria; Firmicutes; Bacilli; Lactobacillales; Streptococcaceae; Streptococcus	0	0	0
OTU _40	Bacteria; Bacteroidetes; Bacteroidia; Bacteroidales; Prevotellaceae; Prevotella	1	0	0
OTU _41	Bacteria; Firmicutes; Bacilli; Lactobacillales; Streptococcaceae; Streptococcus	0	0	0
OTU _46	Bacteria; Firmicutes; Clostridia; Clostridiales; Peptostreptococcaceae; Peptostreptococcus	1	1	0
OTU _81	Bacteria; Fusobacteria; Fusobacteria; Fusobacteriales; Leptotrichiaceae; Leptotrichia	0	0	0
OTU _50	Bacteria; Firmicutes; Negativicutes; Selenomonadales; Veillonellaceae; Veillonella	0	0	1
OTU _25	Bacteria; Fusobacteria; Fusobacteria; Fusobacteriales; Fusobacteriaceae; Fusobacterium	1	3	0
OTU _48	Bacteria; Bacteroidetes; Bacteroidia; Bacteroidales; Prevotellaceae; Prevotella	0	0	0
OTU _134	Bacteria; Firmicutes; Negativicutes; Selenomonadales; Veillonellaceae; Megasphaera	0	0	0
OTU _279	Bacteria; Firmicutes; Bacilli; Lactobacillales; Streptococcaceae; Streptococcus	0	0	0
OTU _425	Bacteria; Firmicutes; Bacilli; Bacillales; Bacillales_Incertae_Sedis_XI; Gemella	0	0	0
OTU _66	Bacteria; Proteobacteria; Gammaproteobacteria; Pasteurellales; Pasteurellaceae; Haemophilus	0	0	1
OTU _60	Bacteria; Firmicutes; Negativicutes; Selenomonadales; Veillonellaceae; Veillonella	2	0	0
OTU _249	Bacteria; Actinobacteria; Actinobacteria; Actinomycetales; Actinomycetaceae; Actinomyces	0	0	0
OTU _257	Bacteria; Proteobacteria; Gammaproteobacteria; Pasteurellales; Pasteurellaceae; Haemophilus	1	0	0
OTU _130	Bacteria; Firmicutes; Negativicutes; Selenomonadales; Veillonellaceae; Veillonella	2	0	0
OTU _210	Bacteria; Firmicutes; Bacilli; Lactobacillales; Streptococcaceae; Streptococcus	0	0	1

OTU _7	Bacteria; Firmicutes; Negativicutes; Selenomonadales; Veillonellaceae; Veillonella	1	1	0
OTU _208	Bacteria; Firmicutes; Bacilli; Lactobacillales; Streptococcaceae; Streptococcus	0	0	2
OTU _39	Bacteria; Fusobacteria; Fusobacteria; Fusobacteriales; Fusobacteriaceae; Fusobacterium	0	1	0
OTU _108	Bacteria; Proteobacteria; Gammaproteobacteria; Pasteurellales; Pasteurellaceae; Haemophilus	0	1	0
OTU _110	Bacteria; Firmicutes; Bacilli; Lactobacillales; Streptococcaceae; Streptococcus	1	2	0
OTU _49	Bacteria; Firmicutes; Negativicutes; Selenomonadales; Veillonellaceae; Veillonella	1	1	0
OTU _92	Bacteria; Firmicutes; Negativicutes; Selenomonadales; Veillonellaceae; Veillonella	0	1	0
OTU _281	Bacteria; Actinobacteria; Actinobacteria; Coriobacteriales; Coriobacteriaceae; Slackia	0	0	0
OTU _214	Bacteria; Firmicutes; Bacilli; Lactobacillales; Carnobacteriaceae; Granulicatella	0	0	1
OTU _116	Bacteria; Firmicutes; Clostridia; Clostridiales; Peptostreptococcaceae; Peptostreptococcus	1	1	0
OTU _13	Bacteria; Firmicutes; Bacilli; Lactobacillales; Streptococcaceae; Streptococcus	0	0	0
OTU _33	Bacteria; Bacteroidetes; Bacteroidia; Bacteroidales; Prevotellaceae; Prevotella	0	0	0
OTU _61	Bacteria; Firmicutes; Negativicutes; Selenomonadales; Veillonellaceae; Veillonella	0	2	0
OTU _16	Bacteria; Firmicutes; Bacilli; Lactobacillales; Streptococcaceae; Streptococcus	0	0	1
OTU _298	Bacteria; Firmicutes; Clostridia; Clostridiales; Lachnospiraceae	0	15	0
OTU _311	Bacteria; Firmicutes; Negativicutes; Selenomonadales; Veillonellaceae; Veillonella	0	0	0
OTU _42	Bacteria; Firmicutes; Negativicutes; Selenomonadales; Veillonellaceae; Veillonella	0	0	0
OTU _83	Bacteria; Firmicutes; Clostridia; Clostridiales; Peptostreptococcaceae; Peptostreptococcus	0	0	0
OTU _124	Bacteria; Bacteroidetes; Bacteroidia; Bacteroidales; Prevotellaceae; Prevotella	0	0	0
OTU _271	Bacteria; Firmicutes; Negativicutes; Selenomonadales; Veillonellaceae; Anaeroglobus	0	0	0
OTU _136	Bacteria; Firmicutes; Negativicutes; Selenomonadales; Veillonellaceae; Veillonella	0	0	0
OTU _31	Bacteria; Firmicutes; Clostridia; Clostridiales; Clostridiales_Incertae_Sedis_XI; Parvimonas	1	1	0

OTU _103	Bacteria; Firmicutes; Bacilli; Lactobacillales; Streptococcaceae; Streptococcus	0	1	0
OTU _135	Bacteria; Firmicutes; Negativicutes; Selenomonadales; Veillonellaceae; Veillonella	0	0	0
OTU _222	Bacteria; Fusobacteria; Fusobacteria; Fusobacteriales; Leptotrichiaceae; Leptotrichia	0	1	0
OTU _64	Bacteria; Firmicutes; Negativicutes; Selenomonadales; Veillonellaceae	2	0	0
OTU _177	Bacteria; Fusobacteria; Fusobacteria; Fusobacteriales; Leptotrichiaceae; Leptotrichia	0	1	0
OTU _30	Bacteria; Fusobacteria; Fusobacteria; Fusobacteriales; Fusobacteriaceae; Fusobacterium	0	1	0
OTU _52	Bacteria; Firmicutes; Negativicutes; Selenomonadales; Veillonellaceae; Veillonella	1	0	0
OTU _418	Bacteria; Bacteroidetes; Flavobacteria; Flavobacteriales; Flavobacteriaceae; Capnocytophaga	0	0	0
OTU _62	Bacteria; Firmicutes; Bacilli; Lactobacillales; Streptococcaceae; Streptococcus	0	0	0
OTU _129	Bacteria; Firmicutes; Bacilli; Lactobacillales; Streptococcaceae; Streptococcus	1	0	0
OTU _441	Bacteria; Bacteroidetes; Flavobacteria; Flavobacteriales; Flavobacteriaceae; Planobacterium	0	1	0
OTU _405	Bacteria; Actinobacteria; Actinobacteria; Actinomycetales; Actinomycetaceae; Actinomyces	0	0	0
OTU _121	Bacteria; Firmicutes; Bacilli; Lactobacillales; Streptococcaceae; Streptococcus	0	0	0
OTU _217	Bacteria; Bacteroidetes; Flavobacteria; Flavobacteriales; Flavobacteriaceae; Capnocytophaga	0	0	0
OTU _175	Bacteria; Firmicutes; Bacilli; Lactobacillales; Streptococcaceae; Streptococcus	0	0	0
OTU _149	Bacteria; Firmicutes; Bacilli; Lactobacillales; Streptococcaceae; Streptococcus	1	1	0
OTU _292	Bacteria; Firmicutes; Bacilli; Lactobacillales; Streptococcaceae; Streptococcus	0	0	0
OTU _91	Bacteria; Firmicutes; Negativicutes; Selenomonadales; Veillonellaceae; Veillonella	0	0	0
OTU _112	Bacteria; Firmicutes; Bacilli; Lactobacillales; Streptococcaceae; Streptococcus	0	0	0
OTU _349	Bacteria; Tenericutes; Mollicutes; Mycoplasmatales; Mycoplasmataceae; Mycoplasma	0	0	0
OTU	Bacteria; Bacteroidetes; Bacteroidia;	1	0	0

_118	Bacteroidales; Prevotellaceae; Prevotella			
OTU _37	Bacteria; Bacteroidetes; Bacteroidia; Bacteroidales; Porphyromonadaceae; Porphyromonas	0	0	0
OTU _313	Bacteria; SR1; SR1_class_incertae_sedis; SR1_order_incertae_sedis; SR1_family_incertae_sedis; SR1_genus_incertae_sedis	0	0	0
OTU _89	Bacteria; Firmicutes; Bacilli; Lactobacillales; Streptococcaceae; Streptococcus	0	0	0
OTU _152	Bacteria; Fusobacteria; Fusobacteria; Fusobacteriales; Fusobacteriaceae; Fusobacterium	1	0	1
OTU _460	Bacteria; Proteobacteria; Alphaproteobacteria; Rhizobiales; Bradyrhizobiaceae; Bradyrhizobium	0	0	0
OTU _335	Bacteria; Fusobacteria; Fusobacteria; Fusobacteriales; Leptotrichiaceae; Leptotrichia	0	0	0
OTU _59	Bacteria; Bacteroidetes; Bacteroidia; Bacteroidales; Prevotellaceae; Prevotella	1	0	0
OTU _138	Bacteria; Bacteroidetes; Bacteroidia; Bacteroidales; Prevotellaceae; Prevotella	0	0	0
OTU _79	Bacteria; Firmicutes; Negativicutes; Selenomonadales; Veillonellaceae; Megasphaera	5	0	0
OTU _122	Bacteria; Firmicutes; Negativicutes; Selenomonadales; Veillonellaceae; Veillonella	1	0	0
OTU _212	Bacteria; Firmicutes; Bacilli; Lactobacillales; Streptococcaceae; Streptococcus	0	0	0
OTU _195	Bacteria; Firmicutes; Bacilli; Lactobacillales; Streptococcaceae; Streptococcus	0	0	0
OTU _219	Bacteria; Firmicutes; Bacilli; Lactobacillales; Streptococcaceae; Streptococcus	0	0	0
OTU _99	Bacteria; Firmicutes; Bacilli; Lactobacillales; Streptococcaceae; Streptococcus	0	0	0
OTU _296	Bacteria; Actinobacteria; Actinobacteria; Actinomycetales; Actinomycetaceae; Actinomyces	0	0	0
OTU _144	Bacteria; Firmicutes; Erysipelotrichia; Erysipelotrichales; Erysipelotrichaceae; Solobacterium	0	1	0
OTU _69	Bacteria; Firmicutes; Negativicutes; Selenomonadales; Veillonellaceae; Veillonella	1	1	0
OTU _334	Bacteria; Bacteroidetes; Flavobacteria; Flavobacteriales; Flavobacteriaceae; Capnocytophaga	0	0	0
OTU _88	Bacteria; Firmicutes; Negativicutes; Selenomonadales; Veillonellaceae; Veillonella	0	0	0

OTU _44	Bacteria; Firmicutes; Clostridia; Clostridiales; Clostridiales_Incertae_Sedis_XI; Parvimonas	0	2	0
OTU _174	Bacteria; Bacteroidetes; Bacteroidia; Bacteroidales; Porphyromonadaceae; Porphyromonas	0	0	0
OTU _101	Bacteria; Firmicutes; Bacilli; Lactobacillales; Streptococcaceae; Streptococcus	0	0	0
OTU _171	Bacteria; Firmicutes; Negativicutes; Selenomonadales; Veillonellaceae; Veillonella	0	0	0
OTU _102	Bacteria; Firmicutes; Erysipelotrichia; Erysipelotrichales; Erysipelotrichaceae; Solobacterium	0	0	0
OTU _117	Bacteria; Firmicutes; Clostridia; Clostridiales; Lachnospiraceae; Oribacterium	0	0	0
OTU _107	Bacteria; Firmicutes; Negativicutes; Selenomonadales; Veillonellaceae; Veillonella	0	0	0
OTU _294	Bacteria; Actinobacteria; Actinobacteria; Coriobacteriales; Coriobacteriaceae; Olsenella	0	0	0
OTU _95	Bacteria; Fusobacteria; Fusobacteria; Fusobacteriales; Fusobacteriaceae; Fusobacterium	0	0	0
OTU _51	Bacteria; Firmicutes; Negativicutes; Selenomonadales; Veillonellaceae; Veillonella	1	0	0
OTU _170	Bacteria; Bacteroidetes; Bacteroidia; Bacteroidales; Prevotellaceae; Prevotella	0	0	0
OTU _356	Bacteria; Firmicutes; Bacilli; Lactobacillales; Aerococcaceae; Abiotrophia	0	1	0
OTU _45	Bacteria; Bacteroidetes; Bacteroidia; Bacteroidales; Prevotellaceae; Prevotella	0	0	0
OTU _310	Bacteria; Firmicutes; Erysipelotrichia; Erysipelotrichales; Erysipelotrichaceae; Solobacterium	1	0	0
OTU _98	Bacteria; Firmicutes; Negativicutes; Selenomonadales; Veillonellaceae; Veillonella	0	0	0
OTU _68	Bacteria; Firmicutes; Negativicutes; Selenomonadales; Veillonellaceae; Veillonella	0	0	0
OTU _104	Bacteria; Firmicutes; Bacilli; Lactobacillales; Streptococcaceae; Streptococcus	1	0	0
OTU _161	Bacteria; Firmicutes; Negativicutes; Selenomonadales; Veillonellaceae; Veillonella	0	1	0
OTU _71	Bacteria; Firmicutes; Bacilli; Lactobacillales; Streptococcaceae; Streptococcus	0	1	0
OTU _264	Bacteria; Firmicutes; Bacilli; Lactobacillales; Carnobacteriaceae	1	0	0
OTU _227	Bacteria; Firmicutes; Negativicutes; Selenomonadales; Veillonellaceae; Veillonella	0	0	0
OTU	Bacteria; Fusobacteria; Fusobacteria;	0	0	0

_114	Fusobacteriales; Fusobacteriaceae; Fusobacterium			
OTU _38	Bacteria; Fusobacteria; Fusobacteria; Fusobacteriales; Fusobacteriaceae; Fusobacterium	0	0	0
OTU _76	Bacteria; Firmicutes; Clostridia; Clostridiales; Lachnospiraceae; Oribacterium	0	0	0
OTU _113	Bacteria; Firmicutes; Bacilli; Lactobacillales; Streptococcaceae; Streptococcus	0	1	0
OTU _55	Bacteria; Bacteroidetes; Bacteroidia; Bacteroidales; Prevotellaceae; Prevotella	0	0	0
OTU _120	Bacteria; Firmicutes; Negativicutes; Selenomonadales; Veillonellaceae; Veillonella	0	0	0
OTU _36	Bacteria; Firmicutes; Clostridia; Clostridiales; Clostridiales_Incertae_Sedis_XI; Parvimonas	0	0	0
OTU _329	Bacteria; Firmicutes; Clostridia; Clostridiales; Peptostreptococcaceae; Peptostreptococcus	0	0	0
OTU _483	Bacteria; Firmicutes; Clostridia; Clostridiales; Lachnospiraceae; Butyrivibrio	0	0	0
OTU _100	Bacteria; Proteobacteria; Epsilonproteobacteria; Campylobacterales; Campylobacteraceae; Campylobacter	1	0	0
OTU _301	Bacteria; Proteobacteria; Gammaproteobacteria; Pasteurellales; Pasteurellaceae	0	1	0
OTU _191	Bacteria; Proteobacteria; Betaproteobacteria; Neisseriales; Neisseriaceae; Eikenella	0	1	0
OTU _297	Bacteria; Firmicutes; Bacilli; Lactobacillales; Streptococcaceae; Streptococcus	0	0	0
OTU _105	Bacteria; Firmicutes; Negativicutes; Selenomonadales; Veillonellaceae	0	0	0
OTU _172	Bacteria; Firmicutes; Bacilli; Lactobacillales	0	0	0
OTU _123	Bacteria; Firmicutes; Negativicutes; Selenomonadales; Veillonellaceae	0	0	0
OTU _157	Bacteria; Firmicutes; Bacilli; Lactobacillales	0	0	0
OTU _96	Bacteria; Firmicutes; Bacilli; Lactobacillales; Streptococcaceae; Streptococcus	0	0	0
OTU _160	Bacteria; Firmicutes; Bacilli; Lactobacillales; Streptococcaceae; Streptococcus	0	0	0
OTU _109	Bacteria; Firmicutes; Negativicutes; Selenomonadales; Veillonellaceae; Veillonella	0	0	0
OTU _206	Bacteria; Firmicutes; Clostridia; Clostridiales; Clostridiales_Incertae_Sedis_XI; Parvimonas	0	1	0
OTU _382	Bacteria; Firmicutes; Clostridia; Clostridiales; Peptococcaceae_1; Peptococcus	0	2	0

OTU _127	Bacteria; Firmicutes; Erysipelotrichia; Erysipelotrichales; Erysipelotrichaceae; Solobacterium	0	1	0
OTU _173	Bacteria; Firmicutes; Negativicutes; Selenomonadales; Veillonellaceae; Veillonella	1	0	0
OTU _193	Bacteria; Firmicutes; Clostridia; Clostridiales; Lachnospiraceae; Oribacterium	0	0	0
OTU _250	Bacteria; Proteobacteria; Epsilonproteobacteria; Campylobacterales; Campylobacteraceae; Campylobacter	0	0	0
OTU _209	Bacteria; Firmicutes; Negativicutes; Selenomonadales; Veillonellaceae; Veillonella	0	0	0
OTU _221	Bacteria; Firmicutes; Clostridia; Clostridiales; Lachnospiraceae; Catonella	0	0	0
OTU _154	Bacteria; Firmicutes; Bacilli; Lactobacillales; Streptococcaceae; Streptococcus	0	0	0
OTU _153	Bacteria; Proteobacteria; Epsilonproteobacteria; Campylobacterales; Campylobacteraceae; Campylobacter	0	0	0
OTU _320	Bacteria; Firmicutes; Negativicutes; Selenomonadales; Veillonellaceae; Megasphaera	0	0	0
OTU _168	Bacteria; Firmicutes; Clostridia; Clostridiales; Lachnospiraceae; Oribacterium	2	0	0
OTU _150	Bacteria; Firmicutes; Clostridia; Clostridiales; Clostridiales_Incertae_Sedis_XI; Parvimonas	0	0	0
OTU _143	Bacteria; Proteobacteria; Epsilonproteobacteria; Campylobacterales; Campylobacteraceae; Campylobacter	1	0	0
OTU _148	Bacteria; Proteobacteria; Epsilonproteobacteria; Campylobacterales; Campylobacteraceae; Campylobacter	0	0	0
OTU _183	Bacteria; Firmicutes; Negativicutes; Selenomonadales; Veillonellaceae; Veillonella	0	0	0
OTU _140	Bacteria; Proteobacteria; Gammaproteobacteria; Pasteurellales; Pasteurellaceae; Mannheimia	2	0	0
OTU _78	Bacteria; Bacteroidetes; Bacteroidia; Bacteroidales; Prevotellaceae; Prevotella	0	1	0
OTU _215	Bacteria; Firmicutes; Clostridia; Clostridiales; Lachnospiraceae; Oribacterium	1	1	0
OTU _289	Bacteria; Firmicutes; Clostridia; Clostridiales; Clostridiales_Incertae_Sedis_XI; Parvimonas	0	0	0
OTU _179	Bacteria; Firmicutes; Bacilli; Bacillales; Bacillales_Incertae_Sedis_XI; Gemella	0	0	0
OTU _317	Bacteria; Proteobacteria; Gammaproteobacteria; Pasteurellales;	0	0	0

	Pasteurellaceae; Mannheimia			
OTU _111	Bacteria; Firmicutes; Negativicutes; Selenomonadales; Veillonellaceae	0	0	0
OTU _285	Bacteria; Firmicutes; Negativicutes; Selenomonadales; Veillonellaceae	1	0	0
OTU _327	Bacteria; Proteobacteria; Gammaproteobacteria; Enterobacteriales; Enterobacteriaceae; Yersinia	1	0	0
OTU _231	Bacteria; Firmicutes; Bacilli; Lactobacillales; Streptococcaceae; Streptococcus	0	0	0
OTU _220	Bacteria; Firmicutes; Negativicutes; Selenomonadales; Veillonellaceae; Veillonella	0	0	0
OTU _236	Bacteria; Fusobacteria; Fusobacteria; Fusobacteriales; Leptotrichiaceae; Leptotrichia	0	0	0
OTU _302	Bacteria; Proteobacteria; Gammaproteobacteria; Pasteurellales; Pasteurellaceae; Mannheimia	0	0	0
OTU _467	Bacteria; Proteobacteria; Alphaproteobacteria; Rhizobiales; Phyllobacteriaceae; Phyllobacterium	0	0	1
OTU _196	Bacteria; Firmicutes; Clostridia; Clostridiales; Lachnospiraceae; Catonella	0	0	0
OTU _345	Bacteria; Firmicutes; Bacilli; Lactobacillales	0	0	0
OTU _230	Bacteria; Bacteroidetes; Bacteroidia; Bacteroidales; Porphyromonadaceae; Porphyromonas	0	0	0
OTU _269	Bacteria; Fusobacteria; Fusobacteria; Fusobacteriales; Fusobacteriaceae; Fusobacterium	0	1	0
OTU _312	Bacteria; Firmicutes; Bacilli; Lactobacillales; Streptococcaceae; Streptococcus	0	0	0
OTU _417	Bacteria; Firmicutes; Negativicutes; Selenomonadales; Veillonellaceae; Dialister	0	0	0
OTU _213	Bacteria; Firmicutes; Negativicutes; Selenomonadales; Veillonellaceae; Veillonella	3	0	0
OTU _234	Bacteria; Firmicutes; Negativicutes; Selenomonadales; Veillonellaceae	0	0	0
OTU _218	Bacteria; Actinobacteria; Actinobacteria; Coriobacteriales; Coriobacteriaceae; Atopobium	0	0	0
OTU _242	Bacteria; Firmicutes; Negativicutes; Selenomonadales; Veillonellaceae; Veillonella	0	0	0
OTU _225	Bacteria; Fusobacteria; Fusobacteria; Fusobacteriales; Leptotrichiaceae; Leptotrichia	0	0	0
OTU _275	Bacteria; Bacteroidetes; Bacteroidia; Bacteroidales; Prevotellaceae; Prevotella	0	0	0
OTU	Bacteria; Firmicutes; Bacilli; Lactobacillales;	0	0	0

_336	Streptococcaceae; Streptococcus			
OTU _344	Bacteria; Firmicutes; Negativicutes; Selenomonadales; Veillonellaceae; Veillonella	0	1	0
OTU _319	Bacteria; Firmicutes; Clostridia; Clostridiales; Eubacteriaceae; Eubacterium	0	0	0
OTU _322	Bacteria; Firmicutes; Negativicutes; Selenomonadales; Veillonellaceae; Megasphaera	0	0	0
OTU _229	Bacteria; Firmicutes; Negativicutes; Selenomonadales; Veillonellaceae; Veillonella	0	0	0
OTU _204	Bacteria; Firmicutes; Negativicutes; Selenomonadales; Veillonellaceae; Megasphaera	1	0	0
OTU _224	Bacteria; Firmicutes; Negativicutes; Selenomonadales; Veillonellaceae; Megasphaera	1	0	0
OTU _338	Bacteria; Bacteroidetes; Bacteroidia; Bacteroidales; Prevotellaceae; Prevotella	0	0	0
OTU _339	Bacteria; Bacteroidetes	0	0	0
OTU _199	Bacteria; Firmicutes; Negativicutes; Selenomonadales; Veillonellaceae; Megasphaera	0	0	0
OTU _325	Bacteria; Firmicutes; Negativicutes; Selenomonadales; Veillonellaceae; Veillonella	0	0	0
OTU _324	Bacteria; Firmicutes; Clostridia; Clostridiales; Clostridiales_Incertae_Sedis_XI; Parvimonas	0	0	0
OTU _192	Bacteria; Proteobacteria; Gammaproteobacteria; Pasteurellales; Pasteurellaceae;	0	0	0
OTU _235	Bacteria; Firmicutes; Negativicutes; Selenomonadales; Veillonellaceae	0	0	0
OTU _351	Bacteria; Firmicutes; Bacilli; Lactobacillales; Streptococcaceae; Streptococcus	0	0	0
OTU _287	Bacteria; Firmicutes; Negativicutes; Selenomonadales; Veillonellaceae; Veillonella	0	0	0
OTU _274	Bacteria; Firmicutes; Clostridia; Clostridiales; Lachnospiraceae; Catonella	0	1	0
OTU _272	Bacteria; Firmicutes; Negativicutes; Selenomonadales; Veillonellaceae; Megasphaera	1	0	0
OTU _260	Bacteria; Firmicutes; Negativicutes; Selenomonadales; Veillonellaceae	0	0	0
OTU _267	Bacteria; Proteobacteria; Epsilonproteobacteria; Campylobacterales; Campylobacteraceae; Campylobacter	1	1	0
OTU	Bacteria; Proteobacteria;	0	0	0

_331	Epsilonproteobacteria; Campylobacterales; Campylobacteraceae; Campylobacter			
OTU _421	Bacteria; Proteobacteria; Alphaproteobacteria; Rhizobiales	0	0	0
OTU _233	Bacteria; Firmicutes; Negativicutes; Selenomonadales; Veillonellaceae; Veillonella	0	0	0
OTU _316	Bacteria; Firmicutes; Clostridia; Clostridiales; Lachnospiraceae; Catonella	0	0	0
OTU _321	Bacteria; Firmicutes; Erysipelotrichia; Erysipelotrichales; Erysipelotrichaceae; Solobacterium	0	0	0
OTU _200	Bacteria; Firmicutes; Erysipelotrichia; Erysipelotrichales; Erysipelotrichaceae; Solobacterium	0	0	0
OTU _270	Bacteria; Firmicutes; Negativicutes; Selenomonadales; Veillonellaceae; Veillonella	0	0	0
OTU _392	Bacteria; Firmicutes; Bacilli; Lactobacillales; Lactobacillaceae; Lactobacillus	0	0	0
OTU _299	Bacteria; Bacteroidetes; Bacteroidia; Bacteroidales; Prevotellaceae; Prevotella	0	0	0
OTU _323	Bacteria; Bacteroidetes; Bacteroidia; Bacteroidales; Prevotellaceae; Prevotella	0	0	0
OTU _471	Bacteria; Actinobacteria; Actinobacteria; Bifidobacteriales; Bifidobacteriaceae; Alloscardovia	0	0	0
OTU _303	Bacteria; Firmicutes; Clostridia; Clostridiales; Lachnospiraceae; Oribacterium	0	1	0
OTU _261	Bacteria; Firmicutes; Negativicutes; Selenomonadales; Veillonellaceae; Centipeda	0	0	0
OTU _248	Bacteria; Bacteroidetes; Bacteroidia; Bacteroidales; Prevotellaceae; Prevotella	0	0	0
OTU _262	Bacteria; Firmicutes; Bacilli; Lactobacillales; Streptococcaceae; Streptococcus	0	0	0
OTU _318	Bacteria; Firmicutes; Clostridia; Clostridiales; Peptostreptococcaceae; Peptostreptococcus	0	0	0
OTU _265	Bacteria; Firmicutes; Negativicutes; Selenomonadales; Veillonellaceae; Veillonella	0	0	0
OTU _342	Bacteria; Proteobacteria; Gammaproteobacteria; Oceanospirillales; Halomonadaceae; Halomonas	0	0	1
OTU _358	Bacteria; Firmicutes; Bacilli; Lactobacillales; Streptococcaceae; Streptococcus	0	0	0
OTU _452	Bacteria; Firmicutes; Bacilli; Lactobacillales; Streptococcaceae; Streptococcus	0	0	0
OTU _295	Bacteria; Firmicutes; Clostridia; Clostridiales; Lachnospiraceae; Oribacterium	0	0	0
OTU	Bacteria; Actinobacteria; Actinobacteria;	0	0	0

_378	Actinomycetales; Micrococcaceae; Micrococcus			
OTU _380	Bacteria; Fusobacteria; Fusobacteria; Fusobacteriales; Fusobacteriaceae; Fusobacterium	1	0	0
OTU _435	Bacteria; Firmicutes; Negativicutes; Selenomonadales; Veillonellaceae; Veillonella	0	0	0
OTU _291	Bacteria; Actinobacteria; Actinobacteria; Coriobacteriales; Coriobacteriaceae; Atopobium	0	0	0
OTU _453	Bacteria; Firmicutes; Bacilli; Lactobacillales; Streptococcaceae; Streptococcus	0	0	1
OTU _429	Bacteria; Firmicutes; Negativicutes; Selenomonadales; Veillonellaceae; Veillonella	0	0	0
OTU _473	Bacteria; Proteobacteria; Gammaproteobacteria; Pasteurellales; Pasteurellaceae; Haemophilus	0	0	0
OTU _394	Bacteria; Firmicutes; Clostridia; Clostridiales; Lachnospiraceae	0	0	0
OTU _459	Bacteria; Firmicutes; Negativicutes; Selenomonadales; Veillonellaceae; Veillonella	0	0	0
OTU _484	Bacteria; Firmicutes; Negativicutes; Selenomonadales; Veillonellaceae	0	0	0
OTU _478	Bacteria; Firmicutes; Clostridia; Clostridiales; Lachnospiraceae	0	0	0
OTU _365	Bacteria; TM7; TM7_class_incertae_sedis; TM7_order_incertae_sedis; TM7_family_incertae_sedis; TM7_genus_incertae_sedis	0	0	0
OTU _420	Bacteria; Bacteroidetes; Bacteroidia; Bacteroidales; Porphyromonadaceae; Porphyromonas	0	0	0
OTU _464	Bacteria; Firmicutes; Negativicutes; Selenomonadales; Veillonellaceae; Veillonella	0	0	0
OTU _413	Bacteria; Firmicutes; Clostridia; Clostridiales; Clostridiales_Incertae_Sedis_XI; Parvimonas	0	0	0
OTU _446	Bacteria; Firmicutes; Bacilli; Lactobacillales; Carnobacteriaceae; Granulicatella	0	0	0

The median OTU-counts per condition of the biofilm grown from pooled saliva are presented with the taxonomy (assigned to the representative sequence of the OTU) as derived from the RDP-classifier/SILVA.
